# Acute effects of blood flow restriction with whole-body vibration on sprint, muscle activation and metabolic accumulation in male sprinters

**DOI:** 10.3389/fphys.2023.1149400

**Published:** 2023-03-16

**Authors:** Junjie Zhang, Ruihang Zhou, Ningning Zhao, Yamei Li, Haiyuan Liu, Wanxia Zhang, Wenxia Guo

**Affiliations:** ^1^ Graduate School, Capital University of Physical Education and Sports, Beijing, China; ^2^ Strength and Conditioning Training Research Center, China Institute of Sport Science, Beijing, China; ^3^ Competitive Sports Research Office, Hebei Institute of Sport Science, Shijiazhuang, China; ^4^ Department of Physical Education, Hebei University of Chinese Medicine, Shijiazhuang, China; ^5^ Guangxi Sports College, Nanning, China; ^6^ Department of Social Sports, Beijing University of Chemical Technology, Beijing, China

**Keywords:** post-activation potentiation, blood flow restriction, vibration training, muscle activation, sprinter

## Abstract

**Purpose:** The aim of this study was to explore the acute effects of Blood Flow Restriction Training (BFRT), Whole-Body Vibration (WBV), and BFRT + WBV on the 20 m sprint, muscle activation, and metabolic accumulation in male sprinters.

**Method:** Sixteen male sprinters randomly performed BFRT, WBV, or BFRT + WBV interventions with 72 h intervals. Electromyography (EMG) signals were collected before and during interventions. Fingertip blood was taken before, immediately after, and 15 min after the intervention. 20 m sprint was performed before and 3 min after the intervention.

**Results:** 1) 0–10m and 0–20 m sprint performance were significantly improved after WBV and BFRT + WBV interventions (*p* < 0.05), 0–20 m sprint performance was significantly improved after all three interventions (*p* < 0.05), 2) After BFRT + WBV intervention, the EMG amplitude of the vastus lateralis and soleus were significantly improved. Greater increases in EMG activity of the tibialis anterior muscle (*p* < 0.05)and blood lactate (*p* < 0.05)were observed following BFRT intervention compared to BFRT + WBV intervention.

**Conclusion:** For sprint performance, BFRT and WBV had similar post-activation enhancement effects to BFRT + WBV, and the metabolic accumulation immediately following the BFRT were higher than that following BFRT + WBV in male sprinters.

## 1 Introduction

Following a conditioning activity (CA), there are significant improvements in the subsequent lower-body explosive performance. The acute increase in muscle performance is known as post-activation potentiation (PAP) (Berriel et al., 2022). Although the exact underlying mechanism for PAP is not fully clear, physiological changes (e.g., muscle temperature, fiber water content, and muscle activation) may contribute to these potential benefits.

Previous studies demonstrated that an appropriate CA could induce significant potential enhancement in jump performance (García-Pinillos et al., 2015). Seven-second maximal voluntary contraction (MVC) intervention significantly improved subsequent vertical jump performance (Robbins and Docherty, 2005). Hoffman et al. (2007) reported that vertical jump performance was significantly improved following back squat intervention in male college rugby players. These acute potential enhancements were also observed in sprint performance (Low et al., 2015). After performing the sled pull sprint exercise, acute improvement in the 15 m sprint was observed in well-trained rugby players (Winwood et al., 2016). Research indicated that body-loaded squat training with whole-body vibration (WBV) significantly increased subsequent on-ice10m and 20 m sprint performance in ice-hockey players (Rønnestad et al., 2016). Notably, Hamada et al. (2003) suggested that to induce ideal potential benefits, the intervention was required to involve high-loading or submaximal-loading. However, high-intensity resistance training also induces acute fatigue, which was related to the increased risk of sports injury and subjective discomfort.

Recently, low-loading resistance training with blood flow restriction training (BFRT) has been proved to induce similar muscle adaptions (e.g., strength and hypertrophy) compared to high-intensity resistance training without BFRT. Thus, this strategy has gained much attention from sports practitioners. BFRT training is a method of restricting blood flow to the intended muscles using special compression devices such as pressure bands and elastic bandages (Scott et al., 2015). Loenneke et al. (2012b) reported that 15%–30%1RM resistance training with BFRT (2,3 times per week) significantly improved muscular strength and muscular hypertrophy, moreover, no significant differences in these improvements were observed between low-intensity with BFRT group and high-intensity resistance training without BFRT. Cook et al. (2014) also demonstrated that 8-week (three times per week) low-intensity resistance training with BFRT training significantly improved 40 m sprint performance and vertical jump height in male rugby players. These findings suggested that short- and long-term low-loading resistance training with BFRT is an effective method to induce positive muscle adaptions.

WBV training is also a popular strength training method as WBV exercise with lower load intensity and shorter intervention duration can significantly improve muscle strength and lower-body explosive performance (Bosco et al., 2000). The underlying mechanisms for WBV training involve the principle of vibratory tension reflex, which induces notable physiological changes in muscle spindle and joint mechanoreceptors (Cook et al., 2014), leading to increasing muscle strength adaptions. Bosco et al. (1999) demonstrated that WBV intervention could significantly increase subsequent muscle power, indicating positive muscle responses to acute WBV intervention. Arora et al. (2021) suggested that WBV significantly increased the neuromuscular activity and the peak power during vertical jump. Furthermore, previous studies reported that WBV intervention with a vibration frequency of 20–50 Hz and an amplitude of 1–12 mm might be an optimal strategy for improving muscle electromyographic responses (Ritzmann et al., 2013; Alam et al., 2018). This finding was in line with the result of the study by Simsek (2017), the author suggested that isometric squat exercise with 40 Hz vibration frequency and 4 mm amplitude could induce activate muscle activity to a greater extent. In this case, adding WBV to low-loading resistance training with BFRT could induce greater muscle activation and motor unit recruitment, resulting in improving subsequent explosive performance compared to resistance training with BFRT alone and resistance training with WBV alone. Unfortunately, little evidence was found in previous studies.

Overall, this study aims to explore the acute effects of BFRT, WBV, and BFRT + WBV interventions on sprint performance, muscle activation, and metabolic accumulation in college male sprinters. We hypothesized that 1) BFRT + WBV intervention would be superior to the BFRT and WBV intervention in inducing increased sprint performance, muscle activation, and metabolic accumulation, and 2) there would be no significant differences in testing variables between BFRT and WBV. The findings of this study will provide alternative strategies for improving sprint performance, *etc.* We will also suggest future research direction.

## 2 Participants and methods

### 2.1 Participants

Participants included in this study were required to meet the following inclusion criteria: 1) elite and sub-elite 100 m or 200 m male sprint athletes; 2) at least 2 years of resistance training experience. 18 male sprinters voluntarily participated in the present study, but only 16 participants complete all tests (mean ± SD; age: 22.93 ± 0.78 years; height: 176.84 ± 3.46 cm; mass: 72.31 ± 4.27 kg; thigh circumference: 56.25 ± 1.59 cm; pressure value of pressure bands: 268.33 ± 20.83 mmHg; training years: 3.40 ± 1.25 years).

All participants should follow all requirements of this study: 1) no high-loading physical activity or consumption of caffeine or alcoholic beverages within 24 h prior to the experiment (Dello Iacono et al., 2018); 2) sufficient intake of water and no food for 4 h before the test (Carbone et al., 2020); 3) no high-intensity training during the experiment. The study has been approved by the ethics committee of blinding for review (2022216H) and all participants signed a consent form.

### 2.2 Procedures

A randomized crossover-controlled design was used in this study. All participants visited the lab four times. The first session was designed to familiarize with the experimental process and isometric squat technique. Also, baseline data including MVC, EMG, blood lactate, and 20 m sprint was collected. In brief, after sitting for 10min, the fingertip blood collection was taken. Then, all participants completed a standardized 5–8 min warm-up session including jogging and dynamic stretch (Figueroa et al., 2012). After a 2–5 min recovery, EMG signals were recorded during MVCs and isometric squat without BFRT and WBV, then participants performed 20 m sprint. All testing programs were performed 3 times, allowing 2–5 min of recovery between tests. In the second to fourth visits, after a standardized warm-up session and a 2–5 min rest interval, all participants randomly performed five sets of 30 s isometric squat exercises (feet keep shoulder-width apart and knees flex to 120°) with a 60s inter-set interval with BFRT or WBV, or BFRT + WBV interventions with 72 h intervals (Dello Iacono et al., 2018; Centner et al., 2019).

EMG was recorded during all three interventions. Moreover, fingertip blood was collected immediately and 15 min following intervention (Contreras et al., 2016; Gómez-Bruton et al., 2017; Centner et al., 2019). Sprint performance was assessed at 3 min post-intervention. During the intervention and testing period, the room temperature was 19.7°C–21.6 °C, and the humidity level was 67%–86%.

### 2.3 Experimental intervention

#### 2.3.1 Blood flow restriction

Participants performed the isometric squat exercise with restricting blood flow. Participants stood upright and the tape was placed at the 2/3 of the long axis between the anterior superior iliac spine and the proximal border of the patella-the top of the patella. When recording the thigh circumference, the tape was snug and horizontal. In this study, the relative pressure method was used to determine the individual exact pressure value of the pressure bands, indicating that the exact pressure of the BFRT bands was selected based on individual thigh circumference. Previous studies have proved that the relative pressure method could induce greater positive muscle adaptions compared with the method of fixed pressure value (Loenneke et al., 2012a; Natsume et al., 2015). Specifically, when the thigh circumference was 45–50cm, 51–55cm, 56–59cm, >60cm, the pressure used was 200 mmHg, 250 mmHg, 300 mmHg, and 350 mmHg, respectively.

Before interventions, participants stood upright with the pressure bands (B Strong, Utah, United States) wrapped around the vertical thigh axis of the transverse gluteus muscle. Considering the training safety, the pressure was gradually increased until the target pressure value was reached. Moreover, in order to gain greater muscle adaptions and decrease the negative effects induced by blood flow restriction, the BFRT intervention was completed within 10min. The average pressure of the blood flow restriction band used was 268.33 ± 20.83 mmHg in this study.

#### 2.3.2 Vibration training

All participants performed the isometric squat exercise when they stood barefoot on a vibration plate (Power Plate pro 5 AIR daptiv, United States) with their feet shoulder-width apart and knees flexed at 120°. In order to measure the knee flexion angle, an electronic goniometer was placed in the right lower limb with one end parallel to the thigh and aligned with the greater trochanter of the femur and the other end parallel to the thigh and aligned with the lateral ankle of the fibula of the calf fibula (Centner et al., 2019). During the BFRT and BFRT + WBV interventions, pressure bands were wrapped to both proximal thighs (Loenneke et al., 2012b). During the WBV, and BFRT + WBV interventions, the vibration plate was set at a frequency of 40 Hz and an amplitude of 4 mm (Figueroa et al., 2012).

### 2.4 Testing

#### 2.4.1 20 m sprint

The 20 m sprint performance was measured using three pairs of timer gates (Swift EZE Jump, Version2.5.28, Brisbane, Australia), which were placed at 0m, 10m, and 20 m. To avoid touching the timer, all athletes started in a three-point position 50 cm behind the start line (Haff and Travis Triplett, 2015). All participants were allowed three attempts with 1–2 min interval time.

#### 2.4.2 EMG

The EMG activity from six muscles of the dominant leg (vastus lateralis (VL), vastus medialis (VM), rectus femoris (RF), tibialis anterior (TA), gastrocnemius medialis (GM), soleus (SOL)) was collected using surface EMG electrodes (Ambu Blue Sensor P, Bad Nauheim, Germany) according to a previous study (Simsek, 2017). Prior to testing, the skin surface was cleaned and wiped with alcohol cotton balls at the VL, VM, RF, TA, GM, and SOL belly positions. The electrodes were placed on the muscle belly of each of the six muscles (Detail information was shown in Table 1) according to the previous studies (Gorsuch et al., 2013; Kazemi et al., 2017; Simsek, 2017) and guidebook (Konrad, 2005). The longitudinal axis of the electrodes followed the orientation of the fibers of the corresponding muscles. All signals are pre-amplified (1,000×), band-pass filtered (10–500 Hz) and sampled at 2 kHz.

**TABLE 1:** Electrode placement.

Muscle	Electrode placement
Vastus lateralis	The electrodes were placed 2/3 of the way on a line from the anterior spina iliaca superior to the lateral side of the patella
Vastus medialis	The electrodes were placed 80% of the way on a line between the anterior spina iliaca superior and the joint space in front of the anterior border of the medial ligament
Rectus femoris	The electrodes were placed at 50% on the line from the anterior spina iliaca superior to the superior part of the patella to record EMG activity
Tibialis anterior	The electrodes were placed one-third of the way between the head of fibula and the medial malleolus of ankle
Gastrocnemius medialis	The quarter-way between the femoral condyles and the calcaneus was used to record the EMG activity
Soleus	The electrodes were placed at 2/3 of the line between the media condylis of the femur to the medial malleolus

Prior to intervention, the EMG signal was recorded when performing the 30s isometric squat (feet keep shoulder-width apart and knees flex to 120°) without BFRT and WBV, and the mean EMG value of the second 10s of all three trials was calculated. Also, the EMG signal was recorded during the MVCs (5s). The MVC test for VL, VM, and RF was according to the study by Roelants et al. (2006), and the MVC test for GM, SOL, and TA was performed following suggestions from Konrad (2005) and Roelants et al. (2006). Both tests were performed 3 times, and the trial showing the highest MVC force was used to further analysis. During all interventions, the EMG signals were recorded during the last three sets of exercise and the mean values were used for analysis.

The raw EMGs for six muscles were converted to the root mean square (RMS) values. To make EMG activity compared among different muscles and participants, the raw EMG signal was normalized to the maximum EMG activity recorded during the MVCs (relative rmsEMG).

#### 2.4.3 Blood lactate

Before the test, a cotton swab dipped in 75% alcohol concentration was wiped on the fingertip, and then blood sample was collected with a blood collection pen, and the first drop of blood was wiped with a clean cotton swab after seeing the blood flow, while 10 mL blood sample was taken and mixed in the lactic acid lysis reagent accompanying the lactic acid analyzer (EKF Biosen C-line, Germany).

### 2.5 Statistical analyses

All data were presented as mean ± standard deviation (M ± SD) and were analyzed using SPSS22.0 (SPSS Inc, Chicago, IL, United States). All variables were examined for normal distribution and homogeneity. The repeated measures analyses of variance (ANOVA) was used to analyze the interaction effects and the Bonferroni correction was used for *post hoc* comparisons. The effect size (ES) was calculated (η^2^), and value between 0.50 and 0.8 represented a medium effect size, value of >0.8 represented a large effect (Cohen, 1992). The significance level was set at *p* < 0.05.

## 3 Results

### 3.1 20 m sprint

**FIGURE 1:**
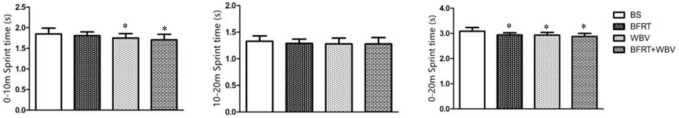
Change in 20 m Sprint time Before and after intervention.

Figure 1 indicates that following interventions, there were no significant differences in 0–10 m (*F* = 1.095, *p* > 0.05), 10–20 m (*F* = 1.451, *p* > 0.05), and 0–20 m (*F* = 0.719, *p* > 0.05) sprint time between BFRT, WBV, and BFRT + WBV. WBV (Pre VS. Post: 1.71 ± 0.05s VS. 1.82 ± 0.18s) and BFRT + WBV (1.69 ± 0.09s VS. 1.82 ± 0.18s) interventions significantly improved the 0–10 m sprint (*p* < 0.05, *ES* > 0.50). BFRT (2.90 ± 0.11s VS. 3.04 ± 0.14s), WBV (2.93 ± 0.11s VS. 3.04 ± 0.14s), and BFRT + WBV (2.85 ± 0.13s VS. 3.04 ± 0.14s) interventions significantly improved the 0–20 m sprint (*p* < 0.05, ES > 0.50).

### 3.2 EMG

**FIGURE 2:**
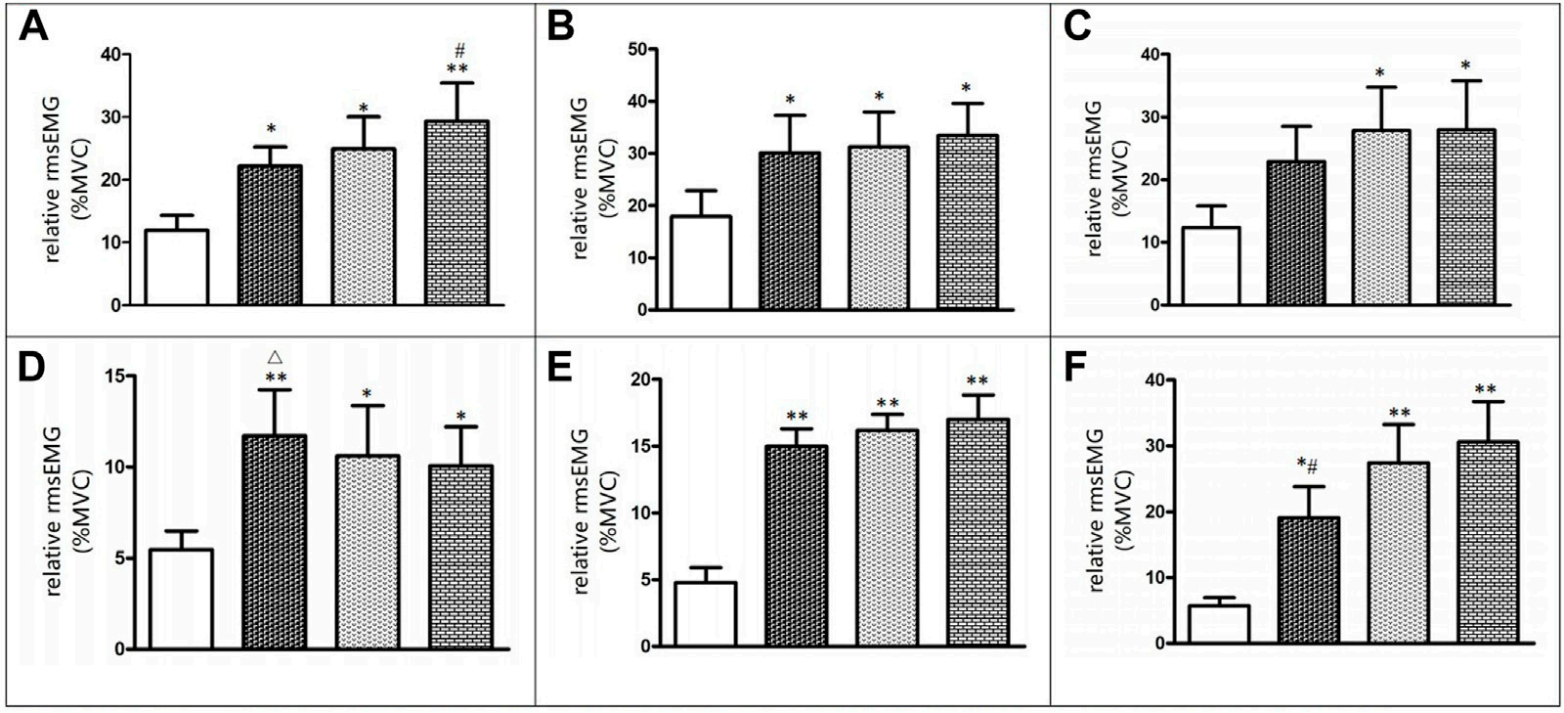
Before and after change in EMG activity level **(A)** Vastus laterails **(B)** Vastus Medials **(C)** Rectus femoris **(D)** Tibialis anterior **(E)** Gastrocnemius medialis **(F)** Soleus.

Figure 2 displays that during interventions, there were significant differences in EMG amplitude of the vastus lateralis (*F* = 4.906, *p* < 0.05), tibialis anterior (*F* = 5.347, *p* < 0.05), and soleus (*F* = 4.573, *p* < 0.05) between BFRT, WBV, and BFRT + WBV. The EMG amplitude of the vastus lateralis, vastus medialis, tibialis anterior, gastrocnemius medialis, and soleus during interventions were significantly greater (*p* < 0.05, *ES* > 0.50) compared to isometric squat without BFRT and/or WBV. Compared to BFRT, BFRT + WBV intervention significantly improved the EMG amplitude of the vastus lateralis and soleus (*p* < 0.05, *ES* > 0.50). Compared to BFRT + WBV, BFRT intervention significantly improved the EMG amplitude of the tibialis anterior (*p* < 0.05, *ES* > 0.50).

### 3.3 Blood lactate

**FIGURE 3:**
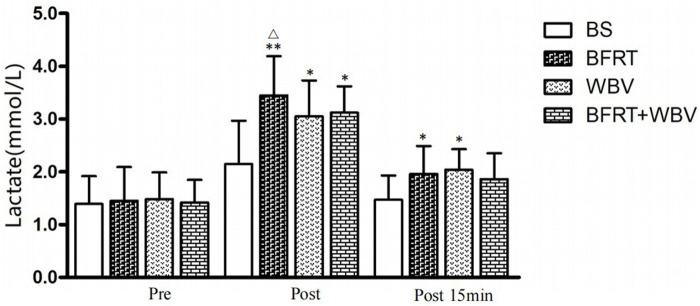
Changes in blood lactate concentration before and after interventions.

Figure 3 shows that there were no significant differences in blood lactate concentration at baseline (*p* > 0.05). However, the significant main effect of time was observed in blood lactate concentrations (*F* = 4.694, *p* < 0.05), and a *post hoc* comparison revealed that blood lactate concentrations were significantly higher after BFRT intervention than BFRT + WBV (*p* < 0.05, *ES* > 0.50). The blood lactate concentration at 15 min after all three interventions did not reach a statistically significant difference (*F* = 0.831, *p* > 0.05), but the blood lactate concentration at 15 min after BFRT and WBV exercises was higher than that of BFRT + WBV, but the difference was not statistically significant (*p* > 0.05).

## 4 Discussion

The main purpose of this study was to investigate the acute effects of BFRT, WBV, and BFRT + WBV interventions on sprint performance, muscle activation, and metabolic accumulation in male sprinters. The sprint time of 0–10m and 0–20 m were significantly improved after WBV and BFRT + WBV interventions, 0–20 m sprint performance was markedly improved after all three interventions. After BFRT + WBV intervention, the increases in EMG amplitude of the vastus lateralis and soleus were significantly greater than after BFRT intervention. Moreover, the EMG amplitude of the tibialis anterior and metabolic accumulations was significantly higher after BFRT intervention than that after BFRT + WBV. These findings indicate that BFRT and WBV can induce similar potential enhancement in sprint performance compared to BFRT + WBV intervention, while BFRT could induce a higher metabolic accumulation than BFRT + WBV immediately after interventions.

All three interventions significantly improved 0–20 m sprint performance. Abe T and Yasuda, (2005) reported that low-loading half squat (20% 1RM) with BFRT significantly improved 0–10m and 0–30 m sprint abilities in the track and field athletes. A study by Manimmanakorn et al. (2013) demonstrated that low-loading resistant exercise combined with BFRT significantly improved 0–5m and 0–10 m sprint abilities in female netball players. Possible explanations for the improvement in sprint performance are related to positive muscle responses, leading to increasing lower-limb power output (Doma et al., 2020; Ienaga et al., 2022). Metabolic accumulation (e.g., lactic acid) caused by restricting blood flow led to cell swell and promotes the secretion of growth hormone, resulting in increases in the muscle cross-sectional area and motor unit recruitment (Takarada et al., 2000). Moreover, our findings are also supported by the results of a study by Rønnestad and Ellefsen (2011), who reported that WBV with a vibration frequency of 50 Hz and an amplitude of 3 mm significantly improved 40 m sprint performance in soccer players. Guggenheimer et al. (2009) suggested that WBV intervention as part of a warm-up session decreased 40 m sprint time by nearly 1/10th of a second. Preconditioning WBV activity has been proved to increase twitch potentiation, the rate of force development, and force and power output (Cochrane et al., 2010). These changes may be explained by increases in phosphorylation of the myosin light chain and/or increased in excitation of involved motor units (Trimble and Harp, 1998). Therefore, these findings suggest that both dynamic and isometric resistance training with BFRT or WBV could significantly improve sprint performance.

Notably, there were no significant differences in sprint performance (0–10 and 0–20 m) following BFRT, WBV, and BFRT + WBV interventions, suggesting that both BFRT and WBV can induce comparable potential benefits in sprint performance. Moreover, significant improvements in sprint performance were also observed following the BFRT + WBV intervention, but adding the BFRT to the WBV intervention has no additional potential benefits to sprint performance. To our best knowledge, no study has explored the acute and long-term effect of BFRT + WBV intervention on sprint performance. The underlying mechanism for this finding is not fully clear and it is also not the scope of this study. We argued that the potential enhancement benefits were influenced by the magnitude of fatigue, which was greater due to the interaction of BFRT and WBV. Moreover, many factors including the recovery level, loading volume, loading intensity, and individual training status also influenced the potential enhancement benefits.

Bordessa et al. (2021) suggested that following BFRT training, quadriceps EMG amplitude was considerably increased in 34 healthy adults. This finding was in line with the present study, we found that BFRT intervention dramatically increased EMG amplitude of the tibialis anterior muscle. Sumide et al. (2009) demonstrated that downhill walking with BFRT increased the EMG amplitude of the soleus muscle, lateral head of the gastrocnemius, and vastus lateralis. The mechanisms for this might be that the oxygen concentration in the muscles is reduced due to restricted blood flow and more muscle fibers or motor units are recruited to perform the intended action. Thus, a greater EMG amplitude was observed. Moreover, hypoxia condition caused by BFRT also enhances phosphocreatine consumption and lower PH value, allowing for the greater activation of type II muscle fibers during exercise (Yasuda et al., 2010).

The BFRT + WBV intervention significantly increased the EMG amplitude of the vastus lateralis, medial head of the gastrocnemius, and soleus muscle, and all three interventions significantly increased the EMG amplitude of the vastus medialis and rectus femoris. Cai et al. (2018) indicated that the EMG values from the rectus femoris and vastus lateralis during the WBV + BFRT session were significantly higher than those during the WBV session (*p* < 0.05). It is reasonable to assume that when BFR is applied with WBV, the primary muscles would have a greater EMG amplitude to accommodate the greater effort, as the blood flow is restricted. Currently, the mechanism of acute WBV remains unclear, but there are two possible explanations for the mediated response of WBV to increased muscle activity. First, the improved EMG amplitude may be due to the fact of a one-to-one firing rate in muscle spindle primary afferents by vibration frequencies up to approximately 70 Hz (Roll et al., 1989). Afferent feedback contributes in reflex to spinal and supraspinal inputs, thereby increasing excitatory influx to the motor neuron pool (Macefield et al., 1993). Thus, it is convinced that WBV with a frequency of 40 Hz may produce muscle spindles to fire at a rate of 40 impulses per second, resulting in a largely increased excitatory stimulation of the motoneuron pool. Second, WBV intervention has been proven to lower the recruitment threshold of the motor unit, especially in high-threshold motor units, and improve the synchronization of the motor units (Romaiguère et al., 1993). Thus, these factors combined may contribute to increased activation of working muscles.

Blood lactate concentration is an important indicator of metabolic accumulation and is influenced by many factors such as mechanical loading, hypoxia, and skeletal muscle involvement. Blood lactate changes are the result of the interaction between the rate of lactate production and the rate of lactate elimination (Cairns, 2006). High lactate tolerance training maximizes the ability of the glycolytic system to supply energy to stimulate the adaptation of the physical system to a certain blood lactate level, improving buffering capacity and muscle lactate dehydrogenase activity, which is directly related to explosive performance. According to the results of this study, peak blood lactate concentrations occurred immediately following the BFRT training intervention, indicating that the glycolysis system could supply energy faster and generate more energy following the BFRT intervention in male sprinters.

Although the mechanisms underlying how increased blood lactate affects neuromuscular activity are unclear, a previous study suggested that metabolic accumulation may facilitate stimulation of group III and IV afferent nerves, thereby inhibiting alpha motor nerve excitability and increasing muscle recruitment to maintain muscle power output (Yasuda et al., 2009), which may provide some support for our findings.

The main limitation of this study. Firstly, the participants in this study were male college sprinters, so the results are not applicable to female, elite, or teenage sprinters. Secondly, the benefits induced by BFRT, WBV, and BFRT + WBV cannot be completely isolated in the present study because of the lack of a control group (isometric squat). Future studies should use a control group in their design. Thirdly, all participants in the present study were required not to participate in heavy physical activities and drink performance-enhancing beverages 48 h before all interventions. In this case, theoretically, no significant difference in sprint performance would occur for all participants. However, sprint performance will fluctuate with changes in mental state. Thus, it is preferable to assess sprint performance before all three interventions respectively.

## 5 Conclusion

Blood flow restriction training alone or vibration training had similar post-activation enhancement effects as blood flow restriction combined with vibration training in sprint running, and the immediate post-activation metabolic accumulation of blood flow restriction training alone was higher than that of blood flow restriction combined with vibration training intervention in the male sprinters.

## Data Availability

The raw data supporting the conclusion of this article will be made available by the authors, without undue reservation.
